# Efficient Inhibition of Hepatitis B Virus Infection by a preS1-binding Peptide

**DOI:** 10.1038/srep29391

**Published:** 2016-07-07

**Authors:** Xiaoli Ye, Ming Zhou, Yonggang He, Yanmin Wan, Weiya Bai, Shuai Tao, Yanqin Ren, Xinxin Zhang, Jianqing Xu, Jing Liu, Junqi Zhang, Kanghong Hu, Youhua Xie

**Affiliations:** 1 Key Laboratory of Medical Molecular Virology (MOE & MOH), Institute of Biomedical Sciences, School of Basic Medical Sciences, Shanghai Medical College, Fudan University, Shanghai, 200032, China; 2 State Key Laboratory of Virology, Institute of Virology, Chinese Academy of Sciences, Wuhan, 430071,China; 3 Shanghai Public Health Clinical Center, Fudan University, Shanghai, 201508, China; 4 Ruijing Hospital, Medical School, Shanghai Jiaotong University, 200025, China; 5 Biomedical Center, Hubei University of Technology, Wuhan, 430068, China

## Abstract

Entry inhibitors are promising novel antivirals against hepatitis B virus (HBV) infection. The existing potential entry inhibitors have targeted the cellular receptor(s). In this study, we aim to develop the first entry inhibitor that inhibits HBV infection via targeting viral particles. The preS1 segment of the large envelope glycoprotein of HBV is essential for virion attachment and infection. Previously, we obtained a preS1-binding short peptide B10 by screening a phage display peptide library using the N-terminal half of preS1 (residues 1 to 60, genotype C). We report here that by means of concatenation of B10, we identified a quadruple concatemer 4B10 that displayed a markedly increased preS1-binding activity. The main binding site of 4B10 in preS1 was mapped to the receptor binding enhancing region. 4B10 blocked HBV attachment to hepatic cells and inhibited HBV infection of primary human and tupaia hepatocytes at low nanomolar concentrations. The 4B10-mediated inhibition of HBV infection is specific as it did not inhibit the infection of vesicular stomatitis virus glycoprotein pseudotyped lentivirus or human immunodeficiency virus type 1. Moreover, 4B10 showed no binding activity to hepatic cells. In conclusion, we have identified 4B10 as a promising candidate for a novel class of HBV entry inhibitors.

More than 350 million people worldwide are chronically infected with hepatitis B virus (HBV)^1^. Current antiviral treatment for chronic hepatitis B includes interferons and nucleos(t)ide analogue reverse transcriptase inhibitors (NRTIs). However, the response rate for interferons is about 20–30% and side effects are common, while the development of drug resistance during long-term therapy with NRTIs is troublesome^2^,^3^. Novel therapeutics acting at different stages of HBV life cycle are in high demand, which, when used alone or combined with current therapies, may improve the antiviral efficacy while in the meantime mitigate or delay the appearance of drug resistance^4^. On the other hand, although envelope protein-based HBV vaccine is effective in protecting a majority of recipients, contraindication or lack of protective response in some recipients calls for alternative prophylactics^5^.

HBV is a small, enveloped, partially double-stranded DNA virus with strict tropism to human hepatocytes. The hepato-tropism is partially attributed to the entry step that presumably begins with reversible, low-affinity attachment of HBV to cell membrane via the interaction between viral envelope proteins and heparan sulfate proteoglycan (HSPG), followed by high-affinity binding of viral envelope proteins to a specific receptor(s) such as the bile-acid pump sodium-taurocholate cotransporting polypeptide (NTCP)^6^,^7^,^8^.

HBV envelope proteins include three membrane glycoproteins, namely large (LHBs), middle (MHBs), and small (SHBs) surface proteins, which are translated from a single open reading frame via different in-frame start codons^9^. SHBs is the major component of viral envelope. MHBs has an extra N-terminal preS2 segment while LHBs, on the basis of MHBs, harbors an additional preS1 segment of 108 to 119 residues depending on different genotypes^6^,^7^. Multiple lines of evidence have indicated that preS1, in particular the N-terminal half and the myristoyl moiety at its amino terminus, is essential for the specific binding of virion to the cellular receptor^6^,^7^,^10^,^11^.

Entry inhibition represents an attractive strategy to prevent the infection and spread of HBV. Myrcludex B, a synthetic N-myristoylated peptide composed of the residues from 2 to 48 of preS1 (preS1/2-48^myr^, genotype D) that harbors the putative receptor binding region could effectively block HBV entry into HepaRG cells, primary tupaia hepatocytes (PTH) and primary human hepatocytes (PHH), as well as inhibit HBV infection in animal models^12^,^13^,^14^. With the identification of NTCP as the cellular receptor of HBV, more molecules have been discovered as potential inhibitors of HBV entry^8^,^15^.

The existing potential entry inhibitors have so far targeted the cellular receptor(s) rather than HBV virion. In this study, we aim to develop the first entry inhibitor that inhibits HBV infection via targeting viral particles. Previously, by screening a phage display peptide library using the N-terminal half of preS1 (residue 1 to 60, genotype C), we obtained a preS1-binding short peptide B10 that could block HBV attachment to HepG2 cells^16^,^17^. We report here that by means of concatenation of B10, we identified a quadruple concatemer designated 4B10 that displayed a markedly increased preS1-binding activity. 4B10 could block HBV attachment to hepatic cells and inhibit HBV infection of primary hepatocytes at low nanomolar concentrations. We further mapped the binding site of 4B10 in preS1 and evaluated the specificity of 4B10-mediated inhibition. The high inhibitory efficacy of 4B10 on HBV infection makes the peptide a promising candidate for a novel class of HBV entry inhibitors.

## Results

### B10 concatemers exhibit increased preS1- and HBV-binding activities

**Figure 1:**
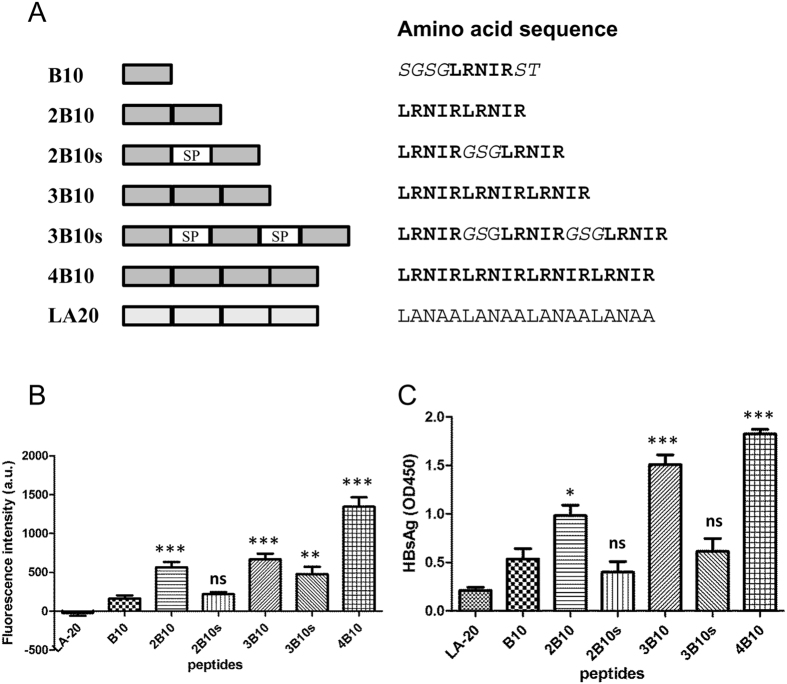
B10 concatemers exhibited increased preS1- and HBV-binding activities. (**A**) Schematic representation of B10 concatemers. B10 monomers were depicted as dark gray boxes (*left*) and in boldface letters (*right*). Terminal protective residues and internal tri-peptide spacers (SP) were *italicized*. (**B**) B10 concatemers exhibit an increased activity in binding to preS1/2-48^myr^. FITC-labelled preS1/2-48^myr^ captured by the immobilized N-biotinylated B10 monomer or concatemer was quantified via fluorescence measurment. (**C**) B10 concatemers exhibit an increased activity in binding to HBV. Captured HBV particles were detected with HRP-conjugated polyclonal anti-HBsAg antibody. *p < *0.05*; **p < *0.01*; ***p < *0.001*; ns, not significant.

A series of N-biotinylated concatemers of the 5-residue B10 were synthesized with or without intervening spacers (Fig. 1A), and their preS1- and HBV-binding activities were evaluated. The lipopeptide preS1/2-48^myr^ was used in preS1-binding assays since it contains the receptor binding region of preS1. Compared to the monomer B10, all the spacerless concatemers displayed a markedly increased activity in binding to preS1/2-48^myr^-FITC (Fig. 1B) and HBV (Fig. 1C). Of all the concatemers, the peptide 4B10 comprising four tandem repeats of B10 showed the highest binding activity. Inclusion of spacers in the concatemers generally had a detrimental effect on preS1-binding activity. The negative control peptide LA-20 displayed no detectable binding activity to preS1/2-48^myr^-FITC and only a very weak one to HBV. Based on the results, 4B10 was chosen for further characterization.

### The main binding site of 4B10 is located within the residues from 28 to 42 of preS1

**Figure 2:**
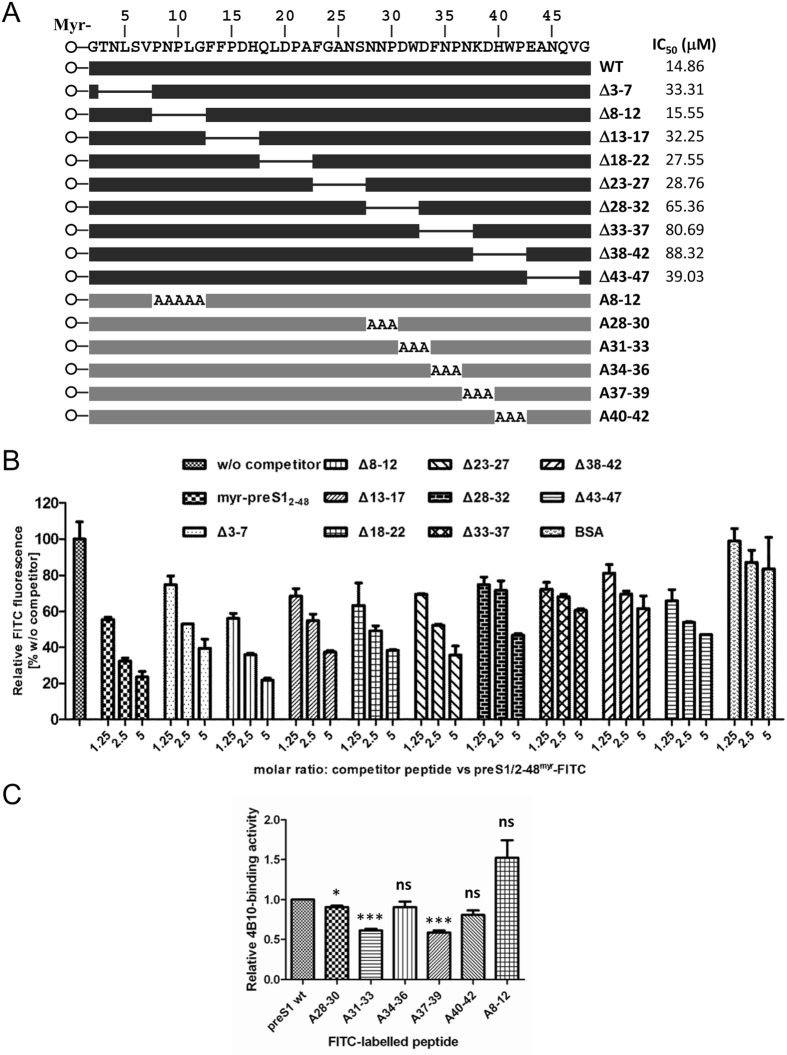
Mapping of the main binding site of 4B10 in preS1. (**A**) Schematic presentation of mutant preS1/2-48^myr^ peptides. IC_50_, concentration for achieving 50% inhibition of the interaction between preS1/2-48^myr^-FITC and 4B10, calculated based on the data plotted in (**B**). The five-amino acid deletions were depicted as lines and the myristoyl groups as open circles. Places where alanine was substituted were indicated. (**B**) Peptide competition assays. Each mutated preS1/2-48^myr^ peptide was used as a competitor of preS1/2-48^myr^-FITC to bind to immobilized N-biotinylated 4B10. The fluorescence intensity was determined and normalized to that from the control competitorless wells. The numbers on the *x* axis represent the molar ratios of competitor versus preS1/2-48^myr^-FITC. BSA, bovine serum albumin. (**C**) 4B10**-**binding activities of alanine substituted preS1/2-48^myr^ peptides. Wild type or alanine substituted preS1/2-48^myr^-FITC was incubated with immobilized N-biotinylated 4B10. The fluorescence intensity was determined and relative binding activity was calculated, with the activity of the wild type peptide taken as 1.0. *p < *0.05*; ***p < *0.001*; ns, not significant.

To determine the binding site of 4B10 in preS1/2-48, a set of mutated preS1/2-48^myr^ peptides containing contiguous 5-amino acid (aa) internal deletions were synthesized (Fig. 2A). Each mutated peptide was premixed with preS1/2-48^myr^-FITC at 1.25-, 2.5-, or 5-fold molar ratio and the mixture was subsequently added to 4B10-coated wells. Non-fluorescent preS1/2-48^myr^ was processed in parallel as a positive control. Any deletion affecting the binding site of 4B10 would theoretically render the corresponding mutated peptide less competitive than preS1/2-48^myr^. As shown in Fig. 2B, all the mutated peptides except Δ8-12 showed more or less reduced competitiveness compared to preS1/2-48^myr^, suggesting that only the deletion of the residues from 8 to 12 did not affect the binding of 4B10 to preS1/2-48^myr^-FITC. The Δ28-32, Δ33-37 and Δ38-42 peptides showed markedly higher IC_50_ (concentration for achieving 50% inhibition) than did others (Fig. 2B), indicating that the residues from 28 to 42 in preS1 constitutes the main binding site of 4B10.

To further map the binding site, we synthesized five mutant preS1/2-48^myr^-FITC peptides with alanine substitution in triplets covering the region 28-42 and a mutant peptide with alanine substitution in residues 8-12 (Fig. 2A). We then investigated their 4B10-binding activities and compared them to that of the wild type peptide. The results showed that alanine substitution in 31-33 or 37-39 effected the most significant reduction in peptide’s 4B10-binding activity (Fig. 2C). In addition, the results suggest that the binding site of preS1/2-48^myr^ in 28-42 is not continuous but rather conformational. Alanine substitution in 8-12 did not result in a reduced 4B10-binding activity of the peptide (Fig. 2C), proving that residues 8-12 is indeed not required for preS1/2-48^myr^’s binding to 4B10.

### 4B10 blocks HBV attachment to hepatic cells

**Figure 3:**
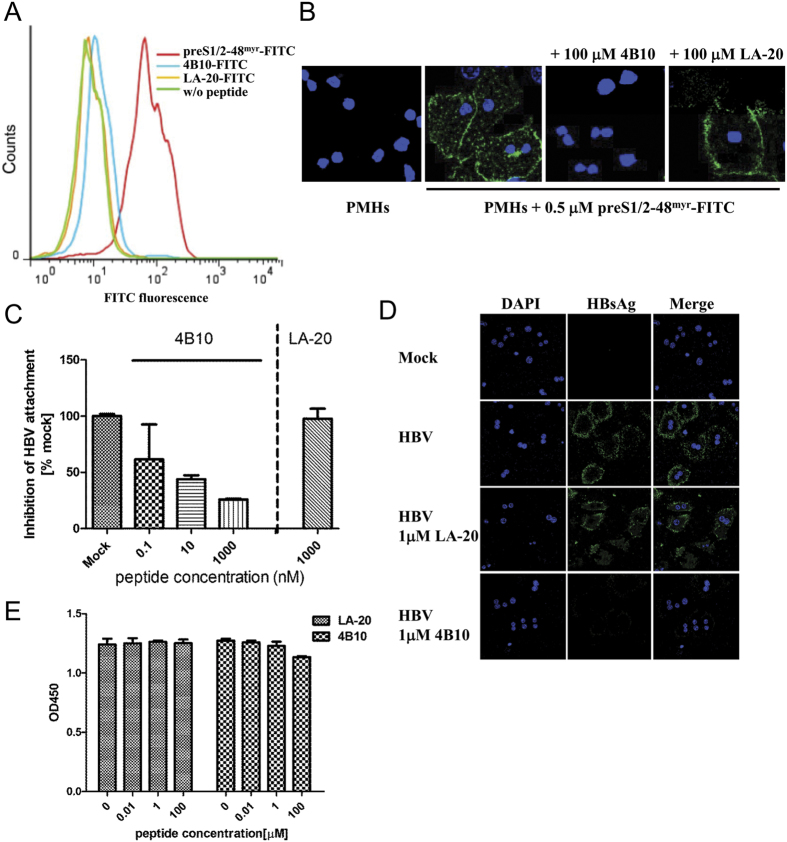
4B10 inhibited HBV attachment to hepatic cells. (**A**) 4B10-FITC and LA-20-FITC did not bind to PMH. Fluorescence-activated cell sorting detected the binding of preS1/2-48^myr^-FITC but not 4B10-FITC and LA-20-FITC to PMH. (**B**) 4B10 inhibited the binding of preS1/2-48^myr^-FITC to PMH. PMH-bound preS1/2-48^myr^-FITC peptides were visualized via confocal fluorescent microscopy. (**C**,**D**) 4B10 inhibited HBV attachment to PMH. (**C**) HBV virions bound to PMH were quantified via real-time PCR. (**D**) Immunofluorescent staining of cell-bound HBsAg. (**E**) 4B10 and LA-20 had no cytotoxic effect on PMH. Cell viability was measured using CCK-8 test.

Specific binding of preS1/2-48^myr^ to primary mouse hepatocytes (PMH) has been documented^18^. Using flow cytometry, we confirmed the binding of preS1/2-48^myr^ to PMH while detected no cellular binding of FITC-labeled 4B10 (4B10-FITC) or LA-20 (LA-20-FITC) (Fig. 3A). We then investigate whether 4B10 might block the cellular binding of preS1/2-48^myr^ and the attachment of HBV. As shown in Fig. 3B, the binding of preS1/2-48^myr^-FITC (0.5 μM) to PMH could be completely blocked by 4B10 applied at a 200-fold molar excess (100 μM) but was barely affected by LA-20 of the same quantity.

We further examined the capability of 4B10 to specifically block HBV attachment to PMH by quantifying PMH-associated virions in the presence of 4B10 or LA-20. 4B10 could dose-dependently reduce the amount of PMH-associated virions (39%, 56% and 74% reductions at 0.1 nM, 10 nM and 1 μM, respectively) while LA-20 (1 μM) exhibited no such effect (Fig. 3C). Immunofluorescent staining of cell-associated surface antigens (HBsAg) also showed that HBV attachment to PMH was markedly reduced in the presence of 4B10 (1 μM) but hardly affected by LA-20 (1 μM) (Fig. 3D). Similar results were obtained with human hepatoma HepG2 cells (Figure S1). To rule out potential cytotoxicity of the peptides, the viability of cells exposed to 4B10 (0.01 μM, 1 μM and 100 μM) or LA-20 for 72 hours was analyzed. No notable cytotoxic effect on PMH or HepG2 cells was observed for both peptides (Figs 3E and S1). Collectively, the results indicate that 4B10 is able to block HBV attachment to hepatic cells.

### 4B10 specifically inhibits HBV infection

**Figure 4:**
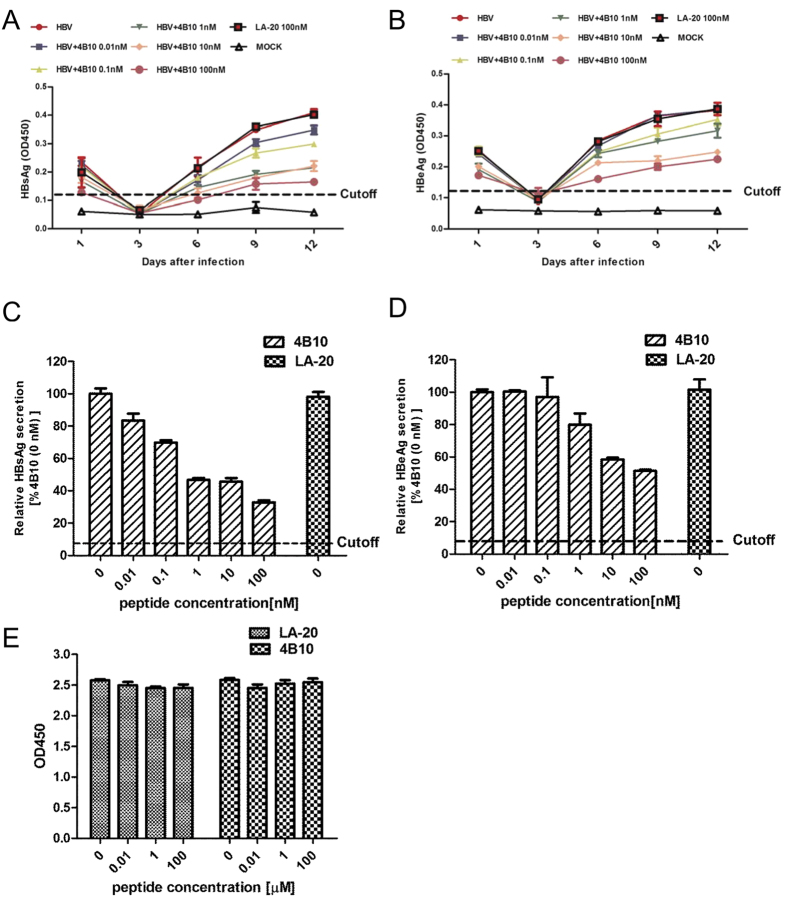
4B10 inhibited HBV infection of primary tupaia hepatocytes . HBsAg (**A,C**) and HBeAg **(B**,**D)** levels in the culture supernatant of PTH infected with HBV preincubated with varying concentrations of 4B10: (**A**,**B**) time course; (**C**,**D**) relative HBsAg and HBeAg levels at day 12 post infection. The HBsAg and HBeAg levels derived from the culture supernatant without 4B10 preincubation were taken as 100% respectively. (**E**) 4B10 and LA-20 had no cytotoxic effect on PTH.

To investigate whether 4B10 inhibits HBV infection, cultured primary tupaia hepatocytes (PTH) and primary human hepatocytes (PHH) were infected with HBV in the presence of 4B10. Newly synthesized and secreted HBsAg and HBeAg by PTH infected with HBV (MOI = 100 GE/cell) could be detected 6 days post-infection and their levels reached a plateau at day 9 to day 12. Pre-incubation with increasing concentrations of 4B10 caused a dose-dependent decrease in post-infection HBsAg and HBeAg expression, while the control peptide LA-20 showed no such effect, indicating a specific inhibition of HBV infection by 4B10 (Fig. 4A,B). The IC_50_ of 4B10 was within the range of 0.1–1 nM based on HBsAg measurement, and 10–100 nM based on HBeAg (Fig. 4C,D).

**Figure 5:**
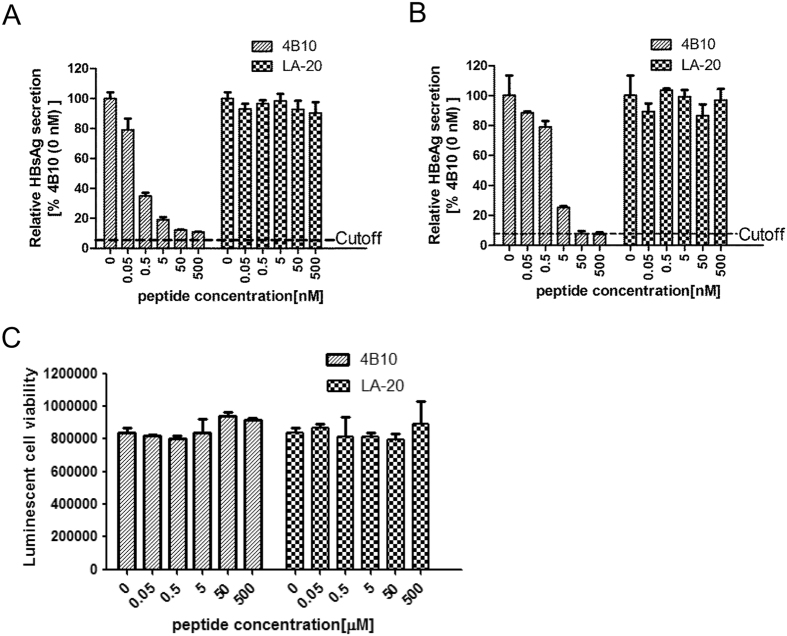
4B10 inhibited HBV infection of primary human hepatocytes. Relative HBsAg (**A**) and HBeAg (**B**) levels at day 14 post infection in the culture supernatant of PHH infected with HBV preincubated with varying concentrations of 4B10. The HBsAg and HBeAg levels derived from the culture supernatant without 4B10 preincubation were taken as 100% respectively. (**C**) 4B10 and LA-20 had no cytotoxic effect on PHH. The proliferation of PHH was measured using CellTiter-Glo Luminescent Cell Viability Assay.

We performed similar assays using cultured PHH. Dose-dependent and efficient inhibition of HBV infection by 4B10 was also evident (Fig. 5A,B). The IC_50_ was about 0.05–0.5 nM and 0.5–5 nM based HBsAg and HBeAg measurement, respectively. No cytotoxicity was observed for 4B10 and LA-20 (Figs 4E and 5C).

**Figure 6:**
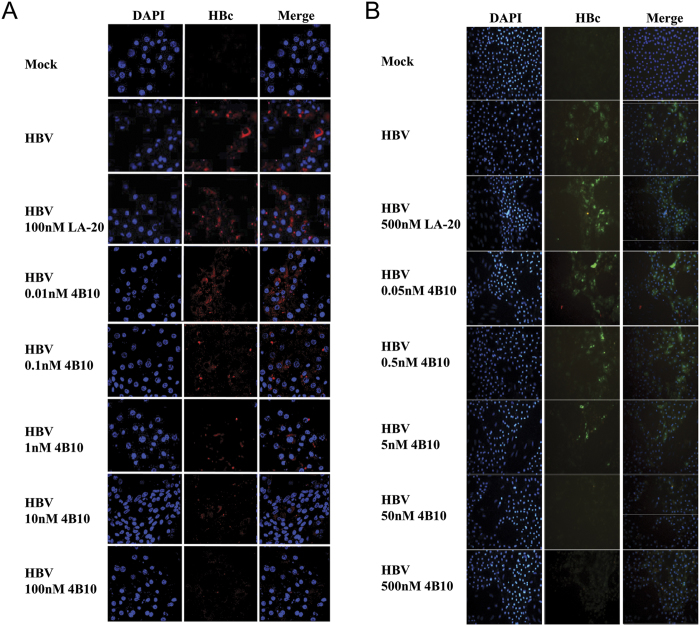
Intracellular HBcAg expression was diminished in PTH and PHH infected with HBV in the presence of 4B10. Intracellular HBcAg in PTH (**A**) and PHH (**B**) at day 12 and day 14 post-infection was detected via immunofluorescence using an anti-HBcAg antibody. Concentrations of peptides pre-incubated with HBV are indicated.

Furthermore, immunofluorescent staining of intracellular viral core protein (HBcAg) clearly demonstrated that in both PTH and PHH, pre-incubation of HBV with 4B10 resulted in a dose-dependent decrease in post-infection HBcAg expression (Fig. 6A,B).

**Figure 7:**
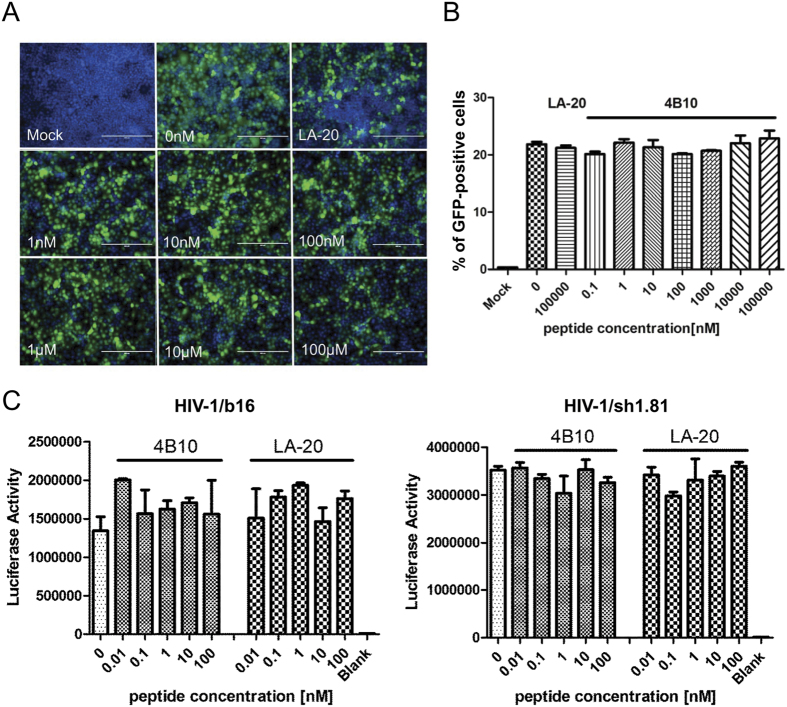
4B10 had no inhibitory effect on the infections of VSV-G pseudotyped lentivirus and HIV-1. (**A**) Huh7 cells were infected with VSV-G pseudotype lentivirus expressing EGFP in the presence of increasing concentrations of 4B10. Percentages of EGFP positive cells were calculated using fluorescence-activated cell sorting and plotted in (**B**). (**C**) TZM-bl cells harboring the HIV-1 LTR-luciferase reporter were infected with replication-deficient HIV-1/b16 (*left*) and HIV-1/sh1.81 (*right*) in the presence of increasing concentrations of 4B10. Intracellular luciferase activities were determined and plotted.

We further examined the specificity of 4B10-mediated inhibition of HBV infection by testing its antiviral activity against the infection of HIV-1 and enhanced green fluorescent protein (EGFP)-expressing vesicular stomatitis virus glycoprotein (VSV-G) pseudotyped lentivirus. Viral infection efficiencies were assessed by measuring Tat-responsive luciferase activity for HIV-1 and live cell EGFP fluorescence for VSV-G pseudotyped lentivirus. Both 4B10 and LA-20 showed no inhibitory effect on the infection of VSV-G pseudotyped lentivirus (Fig. 7A,B) or HIV-1 (Fig. 7C).

## Discussion

The various potential entry inhibitors of HBV infection under development have so far targeted cellular components. The first of their kind, the lipopeptide preS1/2-48^myr^ encompasses the essential region of preS1 required for HBV infection^6^,^10^,^11^,^12^,^13^,^14^. preS1/2-48^myr^ acts as a competitor of HBV by interfering with HBV binding to the cell receptor(s). Synthetic anti-lipopolysaccharide peptides (SALPs) were reported to bind heparan sulfate moieties on cell surface and inhibit infections of not only HBV but also several other enveloped viruses including HCV, HIV-1, HSV-1 and HSV-2 19. This class of HBV entry inhibitors also includes cyclosporin A and some small chemicals that probably affect NTCP’s receptor or transportor activity^20^,^21^,^22^. Targeting cellular components has the notable merit of posing a high barrier to the development of drug resistance, which presumably requires fundamental changes in HBV entry mechanism. Its potential disadvantage, however, is that the physiological functions of the targeted cellular molecules might be affected, particularly during long-term treatment. The peptide 4B10 described in this study targets HBV viral particles by binding to the N-terminal half of preS1 and efficiently inhibits HBV infection. 4B10 itself does not bind to primary hepatocytes or HepG2 cells (Fig. 3A) and displayed no detectable cytotoxicity (Figs 4E and 5C). Therefore, the likelihood of 4B10 interfering with normal hepacellular functions is probably low. Furthermore, 4B10-mediated inhibition of HBV infection is not genotype-dependent as it could inhibit the infection of genotype B and C viruses from HBV-infected patients as well as genotype D viruses derived from HepAD38 cells. More work is required to determine the efficacy of 4B10 against the infection of a broader spectrum of HBV genotypes and clinical mutants.

4B10 might also be an attractive candidate as an alternative prophylactic agent against HBV infection, for example in babies born to HBV-infected mothers, organ transplant recipients, and people nosocomially exposed to HBV. It would be interesting to compare the protective efficacy of 4B10 with that of the currently used human hepatitis B immunoglobulin (HBIG) in animal models. In addition, because 4B10 targets preS1 instead of SHBs, it is possible that 4B10 and HBIG may have a synergistic effect.

Concatemers of B10 without spacers are clearly more potent than the monomer in binding preS1 and HBV (Fig. 1). Concatemerization probably helps the peptide to bring together two or more preS1 domains on the surface of the virion, which may achieve a higher antiviral efficacy. The inclusion of spacers into concatemers generally reduced the peptide’s preS1- and HBV-binding activities. The reason is unknown and might be due to several possibilties. First, spacers may change the binding specificity of the peptide so that it can bind to non-preS1 partners. Second, spacers may make the peptide’s conformation more flexible, especially if the peptide has attached to preS1, which might reduce the preS1-binding affinity of the peptide. Third, the length or the amino acid composition of spacers is perhaps important. We have only used GSG triplet in this study. Redesigning spacers in different length or amino acid combination needs to be explored.

Our results suggest that 4B10 binds to a broad region in preS1/2-48. Nevertheless, the main binding site of 4B10 falls within the residues from 28 to 42 in preS1 and is likely conformational, engaging the residues 31–33 and 37–39. It is noteworthy that the residues from 8 to 12 appeared dispensable for the binding of 4B10 to preS1/2-48. A multi-dimensional NMR study has revealed that preS1 belongs to the family of intrinsically unstructured proteins^23^ and there are several local pre-structured motifs in preS1 likely to be important for viral infection without forming a stable tertiary structure. Previous studies using several HBV infection systems (HepaRG, PTH and PHH) have pointed out that for preS1/2-48^myr^-mediated inhibition of HBV infection, residues from 9 to 18 in preS1/2-48 are essential and those from 29 to 48 are accessory with an enhancing effect, while residues from 19 to 28 is dispensable^24^. Thus, the main binding site of 4B10 matches the accessory domain in preS1/2-48 identified in previous reports. On the other hand, unlike SALPs^19^, 4B10 inhibits HBV infection specifically as it did not inhibit the infection of VSV-G pseudotyped lentivirus or HIV-1, suggesting that the specific interaction between 4B10 and preS1/2-48 as the underlying mechanism. The specificity of 4B10-mediated inhibition needs to be further assessed for more different viruses.

In conclusion, we have identified a preS1-binding peptide 4B10 that specifically inhibits HBV infection. The high inhibitory efficacy of 4B10 and low cytotoxicity make it a promising candidate for a novel class of HBV entry inhibitors.

## Materials and Methods

### Peptide synthesis

All peptides were synthesized using the standard solid-phase synthesis method (GL Biochem, Shanghai, China). preS1/2-48 corresponds to the residues from 2 to 48 of genotype D preS1 or from 13 to 59 of genotype C preS1^6^,^7^. To avoid confusion, we use preS1/2-48 throughout this study. Mutant preS1/2-48 peptides were synthesized, containing a 5-residue internal deletion (Δ3-7, Δ8-12, Δ13-17, Δ18-22, Δ23-27, Δ28-32, Δ33-37, Δ38-42, and Δ43-47) or alanine substitution in 8-12 (A8-12) or alanine substitution in triplet within 28-42 (A28-30, A31-33, A34-36, A37-39, and A40-42) (Fig. 2A). The wild-type and mutant preS1/2-48 peptides were myristoylated at their amino terminus. B10-based peptides and the control peptide LA-20 (Fig. 1A) were N-biotinylated when necessary for immobilization on streptavidin-coated plates (Roche, Basel, Switzerland). The carboxyl termini of preS1/2-48^myr^, alanine substituted preS1/2-48^myr^, 4B10 and LA-20 were conjugated with fluorescein isothiocyanate (FITC) when necessary for fluorescence-based monitoring. Peptide stock solutions were freshly prepared in dimethyl sulfoxide (DMSO) and diluted with PBS or culture medium prior to use.

### Isolation and culture of primary hepatocytes

Primary human hepatocytes were isolated from embryonic livers of post-mortem premature neonates (estimated gestational age, 16–24 weeks), kindly provided by Zhongnan Hospital of Wuhan University with written informed consent from participating family members. PHH were isolated using a modified two-step collagenase perfusion procedure and cultured in William’s E base medium (WEM, Gibco, Grand Island, NY) as described previously^25^,^26^. The ethics and protocol to use tissue samples for this study were approved by the institutional bioethics committee of Wuhan Institute of Virology (registration number: WIVH24201101) in accordance with the principles of the Declaration of Helsinki.

Primary tupaia hepatocytes were prepared and cultured in serum-free medium as described previously^27^. Primary mouse hepatocytes were isolated from 6-week old male C57BL/6 mice with a two-step standard collagenase perfusion protocol and cultured in WEM as previously described^28^. All animal studies were conducted in accordance with protocols approved by the Animal Ethics Committee of Shanghai Medical College at Fudan University (registration number: 20140226-31).

### Cell lines and lentivirus preparation

HepG2, Huh7 and HEK293T cells were maintained in Dulbecco’s modified Eagle medium (DMEM) (Invitrogen, Carlsbad, CA) supplemented with 100 U/ml penicillin G/streptomycin sulfate and 10% (v/v) fetal bovine serum (FBS) (Invitrogen), and cultured at 37 °C with 5% CO_2_. HBV-producing HepAD38 cells^29^ were maintained in DMEM/F-12 medium (Invitrogen) supplemented with 10% FBS and 400 μg/ml G418, and cultured at 37 °C with 5% CO_2_.

Helper plasmid pSPAX2 (Addgene plasmid 12260) and pMD2.G (Addgene plasmid 12259) were co-transfected with pCDH-CMV-MCS-EF1-copGFP (System Biosciences, Mountain View, CA) into HEK293T cells to package VSV-G pseudotyped lentivirus expressing EGFP. Supernatants from co-transfections were used for lentiviral infection.

### preS1/2-48 binding assay and virus capture assay

The N-biotinylated B10 monomer or concatemers (Fig. 1A) (2 μg/well) was applied into the streptavidin-coated wells and incubated for 45 minutes at room temperature. Wells were washed with PBST (0.05% Tween 20 in PBS). FITC-labeled wild type (preS1/2-48^myr^-FITC) or alanine substituted mutant preS1/2-48^myr^ was added and incubated in dark for 1 hour at room temperature. Excess preS1/2-48^myr^-FITC peptides were removed with PBST. Fluorescence intensity was then measured using a VICTOR X multilabel reader (Perkin Elmer, Waltham, MA). In virus capture assays, 1 × 10^6^ viruses from pooled HBV positive serum (Ruijin Hospital, Shanghai) were applied per well. After incubation at 4 °C for 4 hours, wells were washed and 100 μl of horseradish peroxidase-labeled HBsAg antibody (Kehua, Shanghai, China) was added and incubated for 1 hour at room temperature. The absorbance at 450 nm was measured.

For defining the binding site of 4B10 in preS1/2-48, mutated preS1/2-48^myr^ peptide was pre-incubated with 10 μM preS1/2-48^myr^-FITC at 1.25-, 2.5-, or 5-fold molar ratio and added into 4B10-immoblized wells. Co-incubation of preS1/2-48^myr^-FITC with non-fluorescent preS1/2-48^myr^ or bovine serum albumin (BSA) was performed in parallel as positive and negative controls, respectively. Fluorescence was measured as abovementioned. All assays were performed in triplicate on a single plate and repeated at least three times.

### HBV and preS1/2-48myr cell attachment assays

PMH and HepG2 cells were seeded into 12-well plates at a density of 1 × 10^5^ cells per well and cultured in WEM and DMEM without FBS respectively. For preS1/2-48^myr^ binding assays, cells were incubated with 0.5 μM preS1/2-48^myr^-FITC alone or plus 100 μM 4B10 or LA-20 for 4 hours at 37 °C in dark, and washed with ice-cold PBS. Cell nuclei were stained with DAPI (4′6′-diamidino-2-phenylindole) (Sangon, Shanghai, China) for 10 minutes at room temperature. Cells were examined using a confocal microscope (Leica Biosystems, Germany).

For HBV attachment assays, viruses in HepAD38 supernatants were precipitated in 10% polyethylene glycol (PEG) 8000 (Sangon) for 16 hours at 4 °C, centrifuged at 7,000 × g for 1 hour at 4 °C and resuspended in DMEM. Viral titers were determined using a real-time PCR kit (Qiagen, Düsseldorf, Germany) and represented as genome equivalent per milliliter (GE/ml). For peptide-mediated inhibition, 1 × 10^7^ GE/well viruses were pre-incubated with 4B10 (0.1 nM, 10 nM or 1 μM) for 30 min at 37 °C and applied to PMH or HepG2 cells. After incubation at 37 °C for 4 hours, cells were washed repeatedly with ice-cold PBS. Viral DNA was prepared from cell lysate with DNeasy Blood Kit (Qiagen) and quantified using real-time PCR with primers 5′-AATGCCCTATCTTATCAACACT-3′ and 5′-GAGATTGAGATCTTCTGCGACG-3′. All assays were performed in triplicate on a single plate and repeated at least three times.

### HBV Infection assays

Freshly isolated PTH were seeded into collagen-coated 6-well plates (BD Biosciences, San Jose, CA) and maintained in Hepato-Stim Hepatocyte Defined medium (HDM) (BD Biosciences) at 5 × 10^5^ cells per well. Twenty-four hours post-seeding, PTH were infected with concentrated viruses prepared from HepAD38 supernatants (genotype D) at a multiplicity of infection (MOI) of 100 GE per cell. For peptide-mediated inhibition, viruses were pre-incubated with 4B10 (0.01 nM, 0.1 nM, 1 nM, 10 nM or 100 nM) or LA-20 (100 nM) for 30 min at 37 °C in HDM prior to infection. After incubation at 37 °C overnight, cells were washed 5 times with PBS and cultivated for 12 days. For HBV infection of PHH, freshly prepared PHH were seeded into collagen-coated wells in supplemented WEM at 5 × 10^5^ cells per well. Sera from HBV-positive patients (genotypes B and C) were pooled and pre-incubated with 4B10 (0.05 nM, 0.5 nM, 5 nM, 50 nM or 500 nM) or LA-20 for 30 min at 37 °C in WEM pre-mixed with 2% DMSO and 4% PEG 8000, and added three days post-seeding at an estimated MOI of 400. PTH and PHH culture media were changed every two days, and hepatitis B surface antigen (HBsAg) and e antigen (HBeAg) in collected supernatants were determined using enzyme linked immunosorbent assay (ELISA) kits (Kehua). All infections were repeated at least twice.

### Infection with VSV-G pseudotyped lentivirus or HIV-1

VSV-G pseudotyped lentiviruses were pre-incubated with 4B10 (0 nM, 1 nM, 10 nM, 100 nM, 1 μM, 10 μM and 100 μM) or LA-20 (100 μM) for 30 min at 37 °C, applied to Huh7 cells and incubated for 24 hours, then cells were washed with PBS and cultivated for 48 hours post infection. Cells expressing EGFP were visualized under fluorescence microscopy and quantitated using flow cytometry.

The infection of two strains of *tat*-expressing, replication deficient HIV-1, B12 and SH1.81, was tested on TZM-bl cells harboring HIV-1 LTR-luciferase reporter^30^. Envelope proteins of B12 and SH1.81 were derived from HIV-1 B and A/E recombinant subtype, respectively. Briefly, 200 TCID50 (50% Tissue Culture Infective Dose) of B12 or SH1.81 were pre-incubated with 4B10 (0 nM, 1 nM, 10 nM, 100 nM, 1 μM, 10 μM and 100 μM) or LA-20 for 1 hour at 37 °C, and applied to TZM-bl cells. Forty-eight hours post infection, intracellular luciferase activity was measured with a luciferase assay kit (Promega, Madison, WI).

### Immunofluorescence

PHH at 14 days post infection and PTH at 12 days post infection were washed 3 times with ice-cold PBS and fixed in 3.7% paraformaldehyde in PBS for 15 min at room temperature. Cells were washed, permeabilized with 0.5% Triton X-100 in PBS, washed again and incubated in 1% BSA in PBST for 30 minutes at room temperature. Cells were then stained with a polyclonal rabbit anti-HBcAg (1:700 dilution, Dako, Inc, Carpinteria, CA) overnight at 4 °C in a humidified chamber. After washing, PHH and PTH were incubated with Alexa Fluor 488 goat anti-rabbit IgG (H+L) and Alexa Fluor 546 goat anti-rabbit IgG (H+L) (1:1000 dilution, Life Technologies, USA) for 1 hour at room temperature in dark, respectively. After 3 washes in dark, cells were stained with DAPI for 10 minutes at room temperature. For detection of HBV attached to PMH and HepG2 cells, cells were stained with a mouse monoclonal anti-HBsAg (1:1000 dilution, Dako) followed by Alexa Fluor 488 goat anti-mouse IgG (H+L) (1:1000 dilution, Life Technologies). Cells were examined under a confocal microscope (Leica Biosystems).

### Flow cytometry

PTH, PMH, or HepG2 cells (5 × 10^5^ cells/ml) were incubated with 200 nM of preS1/2-48^myr^-FITC, 4B10-FITC or LA-20-FITC in culture medium for 30 min at 37 °C respectively. Cells were washed with PBS containing 0.5% BSA, trypsinized, and re-suspended in PBS. Flow cytometry was performed on a FACS LSRII instrument (BD Biosciences) and analyzed using FlowJo v7.61 (Tree Star, Ashland, OR).

### Cell viability assay

PHH and PTH were seeded into collagen-coated 96-well plates at 5 × 10^4^ cells per well. For PHH, cells were cultured in medium containing 4B10 or LA-20 (0.05 nM to 500 μM) for 24 hours, and viable cells detected using CellTiter-Glo Luminescent Cell Viability Assay (Promega). For PTH, cells were cultured in medium containing 4B10 and LA-20 (0.01 μM, 1 μM or 100 μM) and viability measured using cell counting kit-8 (CCK-8) (Dojindo, Japan).

### Statistical analysis

Student *t* test was used for comparing quantitative variables and *p* value < 0.05 was considered statistically significant.

## Additional Information

**How to cite this article**: Ye, X. *et al.* Efficient Inhibition of Hepatitis B Virus Infection by a preS1-binding Peptide. *Sci. Rep.* **6**, 29391; doi: 10.1038/srep29391 (2016).

## Supplementary Material

Supplementary Information
