# Supplementary material for: Efficient Inhibition of Hepatitis B Virus Infection by a preS1-binding Peptide
Source: Sci Rep. 2016 Jul 7;6:29391. doi: 10.1038/srep29391 (PMC4935942; doi:10.1038/srep29391)

# Efficient Inhibition of Hepatitis B Virus Infection by a preS1-binding Peptide

Xiaoli Ye[1+], Ming Zhou[2+], Yonggang He[1+], Yanmin Wan[3], Weiya Bai[1], Shuai Tao[1], Yanqing Ren[3], Xinxin Zhang[4], Jianqing Xu[3], Jing Liu[1], Junqi Zhang[1*], Kanghong Hu[2, 5*], Youhua Xie[1*]

## Content

**Supplementary figure 1. 4B10 inhibited HBV attachment to HepG2 cells. (A)** HBV virions bound to HepG2 cells were quantified via real-time PCR. **(B)** Immunofluorescent staining of cell-bound HBsAg. **(C)** 4B10 and LA-20 had no cytotoxic effect on HepG2 cells. Cell viability was measured using CCK-8 test.

**Figure S1.**

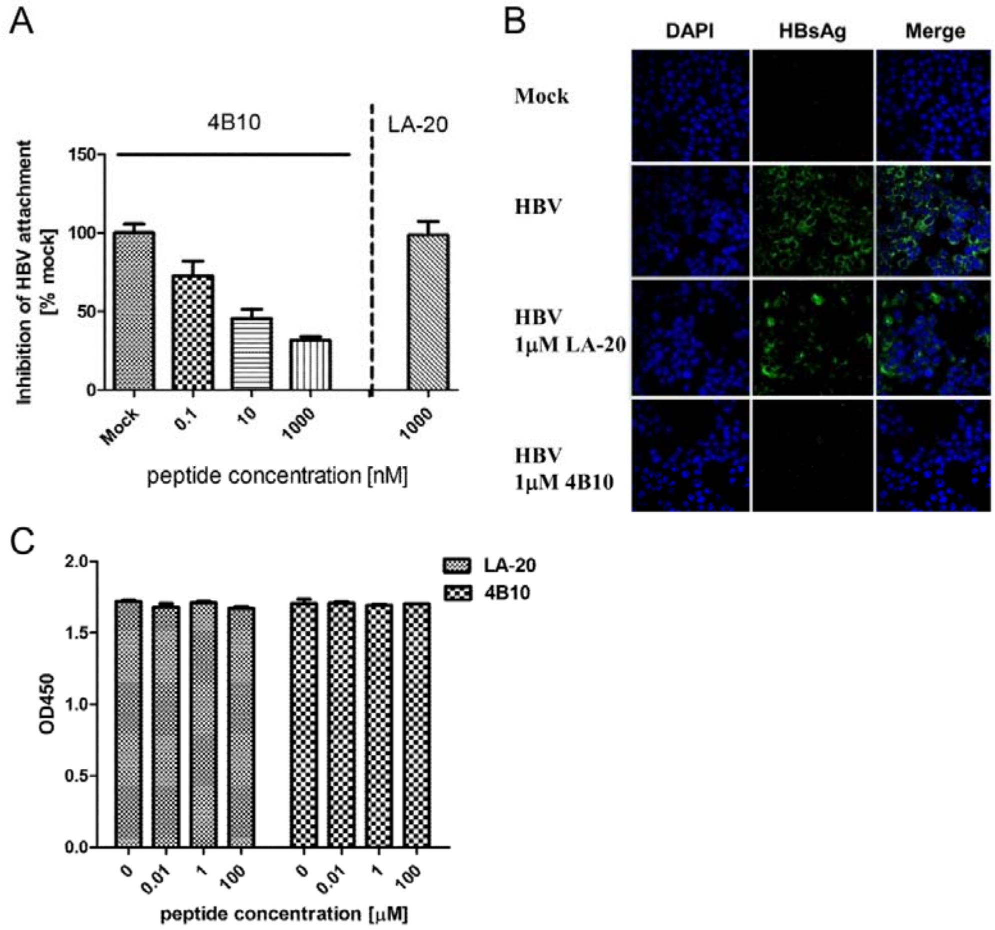

Supplement: Supplementary Information [file srep29391-s1.pdf]
